# Characteristics and long-term health outcomes of the first domestic COVID-19 outbreak cases in Da Nang, Vietnam: a longitudinal cohort study

**DOI:** 10.1186/s41182-024-00670-9

**Published:** 2025-01-14

**Authors:** Miyuki Tsuruoka, Mai Kim Huynh, Michiko Toizumi, Thanh Tien Hoang, Trieu Bao Nguyen, Anh The Dao, Luong Dinh Nguyen, Huy Xuan Le, Chung Thanh Le, Anh Quang Dang, Hung Thai Do, Lay Myint Yoshida

**Affiliations:** 1 Department of Paediatric Infectious Diseases, Institute of Tropical Medicine, Nagasaki University, 1-12-4 Sakamoto, Nagasaki, 852-8523 Japan; 2 Nagasaki University Graduate School of Biomedical Sciences, 1-12-4 Sakamoto, Nagasaki, 852-8521 Japan; 3 Pasteur Institute in Nha Trang, 06-08-10 Tran Phu Street, Nha Trang City, Khanh Hoa Province Vietnam; 4 Nagasaki University School of Tropical Medicine and Global Health, 1-12-4 Sakamoto, Nagasaki, 852-8523 Japan; 5 Centre for Disease Control and Prevention in Da Nang, 315 Phan Chau Trinh Street, Binh Hien Ward, Hai Chau District, Da Nang City, Vietnam

**Keywords:** SARS-CoV-2, COVID-19, Long COVID, Vietnam, Wuhan strain

## Abstract

**Background:**

Vietnam experienced the first COVID-19 domestic outbreak due to the Wuhan strain (B.1.1) in Da Nang from July 2020. COVID-19 can cause acute as well as long-term health problems. We aimed to characterise clinical features and risk factors related to severe illness of COVID-19 among Da Nang outbreak cases and to describe long-term health outcomes among survivors of this outbreak.

**Methods:**

We conducted an ambidirectional cohort study. Study subjects were all hospitalised cases with positive real-time PCR test of SARS-CoV-2 in the three major hospitals in Da Nang from 25 July to 28 August 2020. Clinical and demographic information was retrospectively collected from medical charts. Then, the survivors were followed-up prospectively at 6 and 16 months after acute infection to assess their health status via standardized questionnaires, physical examinations, chest X-rays and pulmonary function tests.

**Results:**

A total of 362 cases including 20 fatal cases were enrolled into the study retrospectively. The median age of the participants included in the medical chart review was 46.5 years and 60.8% were female. Overall, 7.8% of the participants required respiratory support during hospitalisation and 20 of them died. Compared to the survivors, the fatal cases were significantly older (median age of survivors 45.0 yr vs fatal cases 66.5 yr, P < 0.001) and more likely to have underlying conditions. The proportions of participants who had at least one long COVID symptom within the 7 days of each follow-up at 6 and 16 months were 72.0% (134/186) and 63.5% (47/74), respectively. We also found that females and adults reported symptoms more often in the follow-up surveys, 78.9% (90/114) [females] vs 61.1% (44/72) [males] at 6 months, P = 0.008; 68.7% (46/67) [$\ge$ 20 years] vs 14.3% (1/7) $[<$ 20 years] at 16 months, P = 0.004.

**Conclusions:**

In the first domestic COVID-19 outbreak in Vietnam, the mortality rate was approximately 6% and associated with underlying medical conditions. In the follow-up surveys, a substantial proportion of participants reported long COVID related health problems, although the prevalence declined over time. Females and adults reported symptoms more often, which might be due to the pathophysiological differences according to sex and age.

## Background

The emergence of severe acute respiratory syndrome coronavirus 2 (SARS-CoV-2) infections has had a considerable impact worldwide since it started at the end of 2019. Even after recovering from acute infection, an enormous number of people have been debilitated by various symptoms that are considered to be related to SARS-CoV-2 infection and are generally referred to as “long COVID”. The reported symptoms and estimated incidence of those continuing symptoms vary among previous investigations because of the heterogeneity of studies [1–4]. Hypotheses of its pathophysiology have been suggested, which include immune dysregulation, autoimmunity, endothelial dysfunction, occult viral persistence and coagulation activation [5]. However, the mechanisms, risk factors and effective treatments for those symptoms have not been fully elucidated [6].

Vietnam has experienced several big outbreaks of SARS-CoV-2 infections, and the outbreak that occurred in Da Nang from July 2020 was the first domestic outbreak. It started at a major hospital in Da Nang followed by community transmission, resulting in 551 confirmed cases and 35 deaths [7]. Although it was reported that this outbreak was due to the Wuhan strain (B.1.1) [8], there are no detailed reports on the clinical and epidemiological features as well as longitudinal health impacts of this outbreak.

Therefore, we aimed to identify the clinical characteristics and risk factors related to severe illness of COVID-19 in the Da Nang outbreak and to investigate the long-term health outcomes among Da Nang COVID-19 survivors.

## Methods

### Study design and participants

We conducted an ambidirectional cohort study of the SARS-CoV-2 infection cases in the first domestic outbreak in Da Nang, Vietnam, which consisted of a retrospective medical chart review and prospective follow-up surveys. The study subjects were all hospitalised cases with positive real-time polymerase chain reaction (real-time PCR) test of SARS-CoV-2 in three major hospitals in Da Nang (Da Nang Hospital, Da Nang Lung and Respiratory Diseases Hospital, Hoa Vang Hospital), Vietnam, between 25 July and 28 August 2020. This outbreak occurred at the very early stage of the pandemic in Vietnam, where every positive case was traced and all individuals who had contacted positive cases were required to undergo SARS-CoV-2 real-time PCR tests. During this period, individuals who had a positive result for real-time PCR test of SARS-CoV-2 were hospitalised regardless of the presence or absence of symptoms or disease severity in order to contain the spread of SARS-CoV-2.

### Data collection

Firstly, clinical, demographic and socioeconomic information of hospitalised cases was retrospectively collected from medical charts. We used the modified WHO Global COVID-19 Clinical Platform RAPID CORE CASE REPORT FORM, which includes a pregnancy module [9, 10].

Secondly, the survivors were followed-up prospectively at 6 and 16 months after acute infection. We originally planned to conduct the follow-up surveys at 6 and 12 months after acute infection, however we had to change the schedule of the second follow-up due to another COVID-19 outbreak at that timing. In each follow-up survey, participants were interviewed in detail via a standardized questionnaire, physical examinations, pulmonary function tests (PFTs) and chest X-rays to assess their health status. The questionnaire was developed by modifying the International Severe Acute Respiratory and emerging Infection Consortium (ISARIC) follow-up survey form [11], including a series of questions (listed in Figs. 2 and 3) on self-reported symptoms within the 7 days of each follow-up at 6 or 16 months. The questionnaire also included several verified assessment tools, such as the 5-level EuroQol 5-dimension version (EQ-5D-5L) for evaluating health-related quality of life [12]; the Medical Research Council dyspnoea scale (MRC dyspnoea scale) for grading breathlessness from 1 to 5 [13]; the Washington Group Short Set of Questions on Disability (WG-SS) for evaluating problems in daily activities: seeing, hearing, walking, remembering, and self-caring [14]; and the Center for Epidemiologic Studies Depression Scale (CES-D) for measuring depression status [15].

The EQ-5D-5L consists of two parts: (1) the descriptive part asks participants about applicable levels of the five dimensions (mobility, self-care, usual activities, pain/discomfort, and anxiety/depression) and calculates a single summary index for an individual health state according to the EQ-5D-5L value set for Vietnam [16], ranging from − 0.5115 as the worst to 1.0 as the status without any problems on the five dimensions of EQ-5D-5L, and (2) the EQ visual analogue scale (EQ-VAS) rates overall health status based on respondents’ self-perception, ranging from 0 representing the worst imaginable health to 100 representing the best imaginable health [12].

Grades 2–5 on the MRC dyspnoea scale were regarded as dyspnoea. WG-SS grades each activity from 1 to 4 and grades 2–4 were regarded as having a problem. CES-D scores range from 0 to 60, with higher scores indicating greater depressive symptoms; scores of 16 or greater were used as cut-off scores to identify individuals at risk for clinical depression [15]. Those scores were calculated according to the official guidelines for each tool. Furthermore, in the 6-month follow-up survey, the participants were asked about their health status before SARS-CoV-2 infection via the EQ-5D-5L, the MRC dyspnoea scale and the WG-SS as well as the current status.

PFTs and chest X-rays were also implemented in the follow-up surveys. We evaluated the pulmonary functions of the participants based on whether they had “obstructive pattern” defined as FEV1/FVC ratio < 0.70, (FEV1 = forced expiratory volume in one second, FVC = forced vital capacity) or “restrictive pattern” defined as FEV1/FVC ratio ≥ 0.70 and FVC % of predicted < 80%, [17–19].

### Statistical analysis

Demographic and clinical characteristics of the participants in the medical chart review and each of the follow-up surveys were described as medians (interquartile range: IQR) for continuous variables and percentages for categorical variables. For the medical chart review, the Mann–Whitney test was used for continuous variables, and the chi-square test and Fisher’s exact test were used for categorical variables to compare characteristics between alive and fatal cases. To assess the longitudinal health outcomes of the COVID-19 survivors, we described the health status of all participants in the 6- and 16 month follow-up surveys. For the comparison of health outcomes between the different age groups, the sex of the participants and the COVID-19 vaccination status, the Fisher’s exact test and chi square test were used based on the number of people in each group. To analyse the changes in individual health-related quality of life over time, we compared the results of the EQ-5D-5L in the participants who completed both of the follow-up surveys. All the statistical analyses were performed using Stata ver.17.

### Ethics

The study was approved by the Ethical Committee of Nagasaki University (approval number 201021247) and the Pasteur Institute in Nha Trang (approval number 04/IPN-HĐĐĐ). The study was conducted in accordance with the approved guidelines. Written informed consents were obtained from the study subjects or legally acceptable representatives of the participants if the patients died, were minor, or had cognitive difficulties with the chart review and follow-up surveys. No incentive was provided to the participants to participate in the study but a transportation fee of 200,000vnd (~ 8USD) was provided for each follow-up visit to the clinic.

## Results

### Study enrolment

**Fig. 1:**
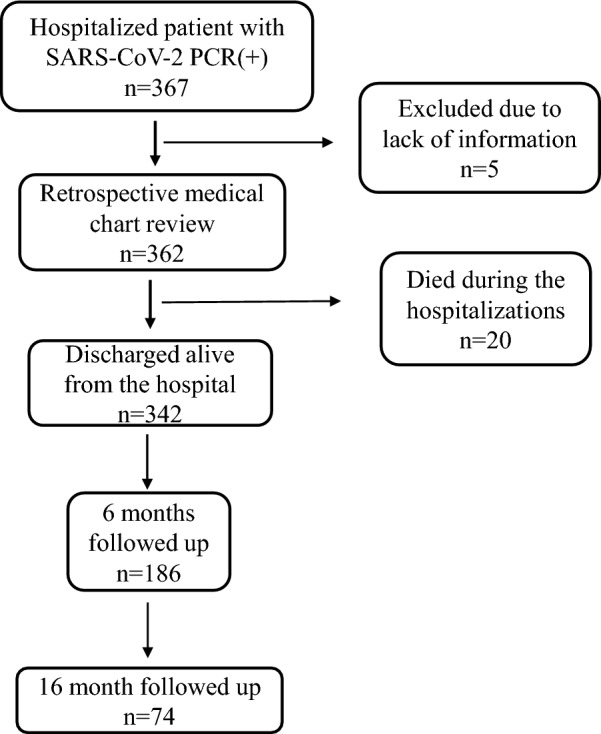
Flow chart of the study

Between 25 July and 28 August 2020, a total of 367 hospitalised cases with positive real-time PCR test of SARS-CoV-2 in the three major hospitals in Da Nang, Vietnam were identified (Fig. 1). Five cases were excluded due to insufficient information and 362 cases were enrolled in the retrospective medical chart review. Out of 362 cases, 20 died during hospitalisation, and all of the 342 survived cases were invited to the prospective follow-up surveys at 6 and 16 months after acute infection. In the 6 month follow-up survey, 186 cases participated, 74 of whom also completed the second follow-up survey at 16 months after infection.

### Medical chart review

#### Characteristics of Da Nang COVID-19 cases (alive and fatal cases)

Among the 362 study participants included in the medical chart review, the median age was 46.5 years (IQR 32–61) and 60.8% (220/362) were female (Table 1). Of 360 cases, who had information on the use of respiratory support, 7.8% (28/360) required respiratory support during hospitalisation, and 20 of them died, resulting in a 5.5% (20/362) of the mortality rate. Compared to the survivors, the fatal cases had a greater median age: 45.0 years (IQR 31–60) [survivors] vs 66.5 years (IQR 52–79) [fatal cases], P < 0.001, and had a significantly greater proportion of underlying conditions such as hypertension: 22.6% (77/341) [survivors] vs 85.0% (17/20) [fatal cases], P < 0.001; chronic kidney disease: 8.2% (28/340) vs 85.0% (17/20), P < 0.001; chronic cardiac disease: 6.8% (23/337) vs 70.0% (14/20), P < 0.001; and diabetes: 8.2% (28/340) vs 31.6% (6/19), P = 0.005. Most of the fatal cases (19 of 20) were infected with SARS-CoV-2 during their hospitalisation for their underlying condition. None of the participants were vaccinated against SARS-CoV-2.

**Table 1:** Underlying characteristics of alive and death cases

Characteristics	Overall		Alive		Death		p-value
	n = 362		n = 342		n = 20		
	n	% or (IQR)	n	% or (IQR)	n	% or (IQR)	
Age	46.5	(32–61)	45.0	(31–60)	66.5	(52–79)	**< 0.001**
Sex							
Male	142	39.2	131	38.3	11	55	0.16
Female	220	60.8	211	61.7	9	45	
Got infected							
During hospitalization	95	26.2	76	22.2	19	95	**< 0.001**
When taking care of family at hospital	42	11.6	42	12.3	0	0	
During working in hospital	35	9.7	35	10.2	0	0	
In community	183	50.6	182	53.2	1	5	
Other	2	0.6	2	0.6	0	0	
Unknown	5	1.4	5	1.5	0	0	
Underlying condition							
Hypertension	94 /361	26	77/341	22.6	17	85	**< 0.001**
Chronic kidney disease	45/360	12.5	28/340	8.2	17	85	**< 0.001**
Chronic cardiac disease	37/357	10.4	23/337	6.8	14	70	**< 0.001**
Diabetes	34/359	9.5	28 /340	8.2	6/19	31.6	**0.005**
Chronic liver disease	6/360	1.7	5/340	1.5	1	5	0.292
Malignant neoplasm	5/360	1.4	3/340	0.9	2	10	**0.027**
Chronic neurological disorder	4/360	1.1	3/340	8.8	1	5	0.205
Pregnant	3/355	0.8	3/340	0.9	0/13	0	> 0.99
Tuberculosis (previous)	2 /360	0.6	2/340	0.6	0	0	> 0.99
Chronic pulmonary disease	1/360	0.3	1/340	0.3	0	0	> 0.99
HIV	1/357	0.3	1/338	0.3	0/19	0	> 0.99
Asthma	0/360	0	0/340	0	0	0	
Current smoking	0/359	0	0/339	0	0	0	
Tuberculosis (active)	0/360	0	0 /340	0	0	0	
Asplenia	0/360	0	0/340	0	0	0	
Treatment before getting COVID-19							
Steroid	8/178	4.5	5/163	3.1	3/15	20	**0.021**
Dialysis regularly	6/170	3.5	3/155	1.9	3/15	20	**0.01**
Anticancer drug	3/177	1.7	1/162	0.6	2/15	13.3	**0.019**
Immunosuppressive drug	0/175	0	0/160	0	0 /15	0	
Severity							
Nonoxygen	312/360	86. 7	312/340	91.8	0	0	
Oxygen therapy	20/360	5.6	20/340	5.9	0	0	
Noninvasive ventilation	2/360	0.6	2/340	0.6	0	0	
Invasive ventilation	22/360	6.1	4/340	1.2	18	90	**< 0.001**
ECMO	4/360	1.1	2/340	0.6	2	10	**< 0.001**
Outcome at discharge							
Discharged alive	322	89	322	94.2	NA	NA	
Hospitalized	8	2.2	8	2.3	NA	NA	
Transferred	12	3.3	12	3.5	NA	NA	
Death	20	5.5	NA	NA	NA	NA	

IQR: interquartile range; HIV: human immunodeficiency virus; ECMO: extracorporeal membrane oxygenation; NA: not applicable

#### Characteristics of the participants in the follow-up surveys

The characteristics of the participants from the follow-up surveys and those of the survivors in the medical chart review were similar in terms of the distributions of age and sex, whereas fewer participants had underlying conditions in the 16-month follow-up survey (Table 2). In both the overall survivor group and the follow-up participants, hypertension was the most common underlying health condition; 22.6% (77/341) [overall survivors], 21.0% (39/186) [at 6 months] and 20.3% (15/74) [at 16 months], followed by diabetes; 8.2% (28/340), 7.0% (13/186) and 5.4% (4/74), chronic kidney disease; 8.2% (28/340), 4.8% (9/186) and 2.7% (2/74), or chronic cardiac disease; 6.8% (23/337), 5.4% (10/186) and 2.7% (2/74). Most of the participants in the follow-up surveys were asymptomatic or had mild symptoms in the acute phase that did not require oxygen supply or respiratory support during the hospitalisation, with 94.6% (176/186) and 97.3% (71/73) of the participants in the 6- and 16-month follow-up surveys, respectively.

**Table 2:** Underlying characteristics of overall survivors and participants of follow‐up surveys at 6 and 16 months

Characteristics		Overall survivors		At 6 months		At 16 months	
		n = 342		n = 186		n = 74	
		n	% or (IQR)	n	% or (IQR)	n	% or (IQR)
Age (year, median)		45	(31–60)	46	(30–59)	48.5	(35.6–60)
Sex	Male	131	38.3	72	38.7	29	39.2
	Female	211	61.7	114	61.3	45	60.8
Got infected	During Hospitalization	76	22.2	26	14.0	9	12.1
	While caring family at the hospital	42	12.3	14	7.5	6	8.1
	During working in the hospital	35	10.2	15	8.1	6	8.1
	In communities	182	53.2	124	66.7	49	66.2
	Other	2	0.6	4	2.2	3	4.1
	Unknown	5	1.5	3	1.6	1	1.4
Underlying conditions	Hypertension	77/341	22.6	39	21.0	15	20.3
	Diabetes	28/340	8.2	13	7.0	4	5.4
	Chronic kidney disease	28/340	8.2	9	4.8	2	2.7
	Chronic cardiac disease	23/337	6.8	10	5.4	2	2.7
	Chronic liver disease	5/340	1.5	3	1.6	0	0
	Malignant neoplasm	3/340	0.9	2	1.1	0	0
	Chronic neurological disorder	3/340	0.9	1	0.5	0	0
	Tuberculosis (previous)	2/340	0.6	2	1.1	1	1.4
	HIV (not on ART)	1/338	0.3	1	0.5	0	0
	Chronic pulmonary disease	1/340	0.3	1	0.5	0	0
	Asthma	0/340	0	0	0	0	0
	Current smoking	0/339	0	0	0	0	0
	Tuberculosis (active)	0/340	0	0	0	0	0
	Asplenia	0/340	0	0	0	0	0
Severity	Non-oxygen	312/341	91.5	176	94.6	71/73	97.3
	Oxygen therapy	21/341	6.2	9	4.8	2/73	2.7
	Non-invasive ventilation	2/341	0.6	0	0	0	0
	Invasive ventilation	4/341	1.2	1	0.5	0	0
	ECMO	2/341	0.6	0	0	0	0
Outcome at discharge	Discharged alive	322	94.2	182	97.8	73	98.6
	Hospitalized	8	2.3	3	1.6	0	0
	Transferred	12	3.5	1	0.5	1	1.4
COVID-19 vaccination status at 16 months*	0 dose	NA	NA	NA	NA	0	0
	1 dose	NA	NA	NA	NA	9	4.0
	2 doses	NA	NA	NA	NA	60	81.1
	Unknown	NA	NA	NA	NA	5	2.0

^ECMO: extracorporeal membrane oxygenation; HIV: human immunodeficiency virus; ART: active antiretroviral therapy^

^*COVID-19 vaccination programme began in Vietnam between the 1st and 2nd follow-up survey.^

In March 2021, Vietnam launched its COVID-19 vaccination programme by first providing vaccinations to health care workers. In the second follow-up survey, conducted in November 2021, 85.1% (69/74) of the participants reported that they had received COVID-19 vaccinations (81.1% received two doses and 4.0% one dose).

#### Physical symptoms reported in the follow-up surveys

**Fig.2:**
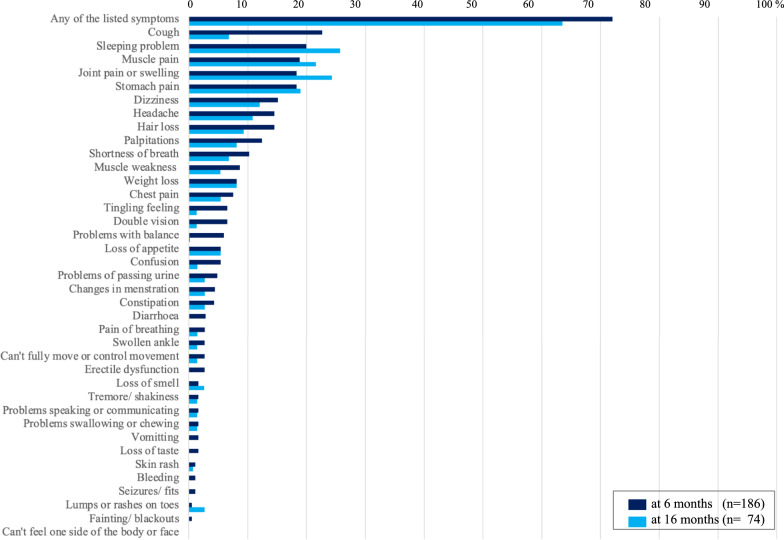
The symptom prevalence at 6 and 16 months after acute infection. The bar graphs indicate the prevalence (%) of symptoms listed in the questionnaire for the follow-up surveys at 6 and 16 months after acute infection with SARS-CoV-2

**Fig. 3:**
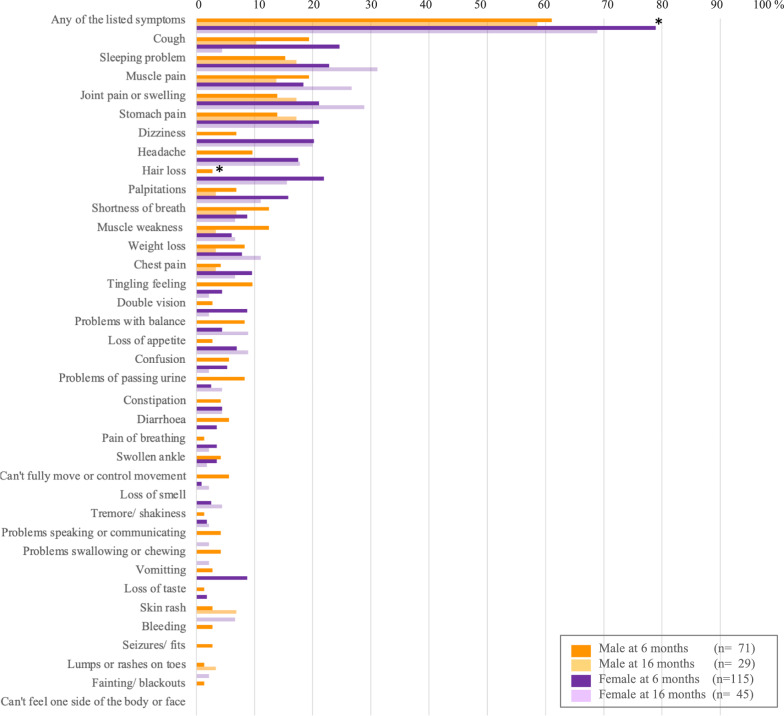
The symptom prevalence by sex at 6 and 16 months after acute infection. The bar graphs indicate the prevalence (%) of symptoms listed in the questionnaire for the follow-up surveys at 6 and 16 months after acute infection with SARS-CoV-2 by sex. Statistical analyses for comparisons of symptom prevalence by sex at each follow-up survey were conducted via the chi-square test or Fisher’s exact test. *Compared to males, a significantly greater proportion of females reported any of the listed symptoms in the questionnaire at 6 months (P = 0.008) and hair loss at 6 months (P < 0.001)

The proportions of participants who had at least one symptom asked in the questionnaire (listed in Figs. 2 and 3) within the 7 days of each follow-up at 6 or 16 months were 72.0% (134/186) and 63.5% (47/74), respectively (Fig. 2). The most common symptoms reported in the follow-up survey at 6 months were cough (22.6%, 42/186), sleeping problems (19.9%, 37/186), persistent muscle pain (18.8%, 35/186), joint pain or swelling (18.3%, 34/186) and stomach pain (18.3%, 34/186). While cough was the most common symptom in the 6 month survey, its prevalence decreased markedly in the 16 month survey (6.8%, 5/74). On the other hand, the prevalence of several symptoms, such as sleeping problems (25.7%,19/74), persistent muscle pain (21.6%, 16/74), and joint pain or swelling (24.3%, 18/74), increased at 16 months. The proportion of females who had one or more symptoms at the 6 month follow-up was significantly greater than that of males: 78.9% (90/114) [females] vs 61.1% (44/72) [males], P = 0.008 (Fig. 3). In addition, the prevalence of hair loss in females at 6 months was significantly greater than that in males: 21.9% (25/114) [females] vs 2.8% (2/72) [males], P < 0.001. In the 16 month follow-up survey, although not significant, the proportion of females who had at least one symptom was greater than that of males, 68.9% (31/45) [females] vs 58.6% (17/29) [males], P = 0.366. Comparing the results of the 6 month and 16 month surveys, the prevalence of some symptoms increased among females, such as sleeping problems, from 22.8% (26/114) to 31.1% (14/45); persistent muscle pain, from 18.4% (21/114) to 26.7% (12/45); joint pain or swelling, from 21.1% (24/114) to 28.9% (13/45); and weight loss, from 7.9% (9/114) to 11.1% (5/45), although the prevalence of most symptoms among males decreased.

In both of the follow-up surveys, the proportion of adult people who had any symptoms was greater than that of younger people at 6 months: 73.3% (121/165) [$\ge$ 20 years] vs 61.9% (13/21) [< 20 years], P = 0.272; at 16 months, 68.7% (46/67) [$\ge$ 20 years] vs 14.3% (1/7), [< 20 years] P = 0.004). Among 45 participants who reported at least one symptom in the 16 month survey, 41 (91.1%) had received two doses of COVID-19 vaccine, and 4 (8.9%) had received one dose. There was no significant difference in symptom prevalence according to the number of COVID-19 vaccinations (P = 0.259).

#### Changes in self-impressions of overall health status and health-related quality of life

When comparing between 6- and 16 month follow-ups, the proportion of participants who did not experience fatigue increased from 49.4% (89/186) to 61.4% (43/70), and the proportion of those who believed “fully recovered from COVID-19” increased from 82.3% (153/186) to 91.8% (67/73). In addition, 80% (56/70) of the participants had returned to work at 16 months after infection (Table 3). In the 16 month survey, six participants (8.2%) reported that they did not fully recover from COVID-19. The median age of the six cases was 55.5 years, and four (66.7%) were female. The common symptoms at 16 months after infection were headache, shortness of breath, and sleeping problems. Before contracting COVID-19, five participants had no problems with the EQ-5D-5L, however, three participants reported some problems at the 16 month follow-up. One of them reported problems in all five dimensions of the EQ-5D-5L: mobility, self-care, usual activities, pain/discomfort, and anxiety/depression. The other two participants had problems with the two dimensions of the EQ-5D-5L, pain/discomfort and anxiety/depression, and one of them was diagnosed with depression and arthritis by a physician in the 16-month survey.

**Table 3:** Health outcomes of COVID-19 survivors in the 6- and 16-month follow-up surveys

	Prevalence of participants				
	in the 6 month survey (n =186)		in the 16 months survey (n = 74)		
	Before COVID-19	At 6 months	Before COVID-19	At 6 months	At 16 months
EQ-5D-5L					
Anxiety or depression	26 (14.0%)	42 (22.6%)	11 (14.9%)	20 (27.0%)	5/58 (8.6%)
Pain or discomfort	19 (10.2%)	33 (17.7%)	7 (9.5%)	17 (23.0%)	4/58 (6.9%)
Mobility problem	11 (5.9%)	11 (5.9%)	3 (4.1%)	3 (4.1%)	3/58 (5.2%)
Usual activity problem	9 (4.8%)	11 (5.9%)	3 (4.1%)	4 (5.4%)	3/58 (5.2%)
Self-care problem	8 (4.3%)	8 (4.3%)	3 (4.1%)	2 (2.7%)	2/58 (3.4%)
EQ-5D-5L Value for all health*					
< 1.0	34 (18.3%)	56 (30.1%)	14 (18.9%)	21 (28.4%)	7/58 (12.1%)
EQ-VAS scale: 0–100					
Median (IQR)	NA	90 (80–90)	NA	80 (75–90)	85 (80–90)
CES-D (depression scale: 0–60)					
≧16 (Depression)	NA	22 (11.8%)	NA	9 (12.1%)	11 (14.9%)
WG-SS					
Walking	41/184 (22.3%)	46/184 (25.0%)	14 (18.9%)	17(23.0%)	18/72 (25.0%)
Remembering/concentrating	39 (21.1%)	55 (29.7%)	18 (24.3%)	23 (31.1%)	32/71(45.1%)
Seeing	33 (17.8%)	47 (25.4%)	15 (20.3%)	22 (29.7%)	26 (36.1%)
Hearing	7 (3.8%)	11 (5.9%)	2 (2.7%)	2 (2.7%)	10/72 (13.9%)
Self-care	6 (3.2%)	8 (4.3%)	2 (2.7%)	4 (5.4%)	2/72 (2.8%)
Language use	2 (1.1%)	4 (2.2%)	1 (1.4%)	2 (2.7%)	1/72 (1.4%)
Self-impressions					
Not fatigue	NA	89/186 (49.4%)	NA	42/73 (57.5%)	43/70 (61.4%)
Fully recovered	NA	153/186 (82.3%)	NA	59 (79.7%)	67/73 (91.8%)
Return to original work	NA	159/186 (85.5%)	NA	63 (85.2%)	56/70 (80.0%)
Chest X-ray					
Abnormal	NA	28/184 (15.2%)	NA	13/72 (18.1%)	4/70 (5.7%)
Pulmonary function test**					
Obstructive pattern	NA	11/178 (6.2%)	NA	4/70 (5.7%)	7/68 (10.3%)
Restrictive pattern	NA	66/178 (37.1%)	NA	28/70 (40.0%)	23/68 (33.8%)
MRC dyspnoea scale					
1	90/108 (83.3%)	79/100 (79.0%)	36/42 (85.7%)	31/38 (81.6%)	10/11 (90.9%)
≧ 2 (dyspnoea)	18 (16.7%)	21/100 (21.0%)	6/42 (14.3%)	7/38 (18.4%)	1/11 (9.1%)

Health outcomes over time was assessed with health-related quality of life (EQ-5D-5L: 5-level EQ-5D Questions), overall health status (EQ-VAS: EQ visual analogue scale),depression scale (CES-D: Center for Epidemiologic Studies Depression Scale), activity limitations (WG-SS: The Washington Group Short Set of Questions on Disability), self-impressions, pulmonary function test, chest X-ray and dyspnoea (MRC dyspnoea scale)

*The Vietnam values for all health by EQ-5D-5L range from − 0.5115 to 1 [13]

**Pulmonary function test, Obstructive pattern: FEV1 (forced expiratory volume in one second)/FVC (forced vital capacity) ratio < 0.70, Restrictive pattern: FEV1/FVC ≧ 0.70 and FVC % of predicted < 80%. NA: not applicable

With respect to health-related quality of life, as assessed with the EQ-5D-5L, among the participants who completed the both follow-ups at 6 and 16 months, the proportions of participants who had problems with “anxiety or depression” or “pain or discomfort” and selected any level “2–5” in these two dimensions, increased at 6 months compared to before getting COVID-19, from 14.9% (11/74) to 27.0% (20/74) and from 9.5% (7/74) to 23.0% (17/74), respectively. However, it decreased at 16 months, 8.6% (5/58) [anxiety/depression] and 6.9% (4/58) [pain/discomfort] (Table 3). For the value for all health, which was calculated with the EQ-5D-5L value set for Vietnam [16], the proportion of participants whose value was less than 1.0 increased from 18.9% (14/74) before contracting COVID-19 to 28.4% (21/74) in the 6-month survey. However, that percentage decreased to 12.1% (7/58) in the 16-month survey. Additionally, the median EQ-VAS score in the 16-month survey, 85 (IQR 80–90), was greater than that in the 6-month survey, 80 (IQR 75–90) (Table 3).

#### Chest X-rays and PFT

The proportion of participants with abnormal chest X-rays decreased from 15.2% (28/184) at 6 months to 5.7% (4/70) at 16 months (Table 3). All four abnormal chest X-rays at the 16 month follow-up revealed pulmonary fibrosis. For PFTs, the proportion of participants indicating restrictive patterns decreased from 37.1% (66/178) at 6 months to 33.8% (23/68) at 16 months, whereas the proportion of participants with obstructive patterns slightly increased from 6.2% (11/178) to 10.3% (7/68) (Table 3). There were three participants whose pulmonary function clearly deteriorated at the 16 month follow-up compared to the 6 month follow-up. None of these three patients required oxygen or respiratory support during their hospitalisations for COVID-19, and none of them smoked. One patient had a history of tuberculosis, and he was diagnosed with suspected COPD (chronic obstructive pulmonary disease) in the 16 month survey. Another subject had hypertension before contracting COVID-19, was diagnosed with diabetes after contracting COVID-19, and experienced a heart attack between the follow-ups. A pulmonary function test revealed a restrictive pattern in the 16 month follow-up survey. The other subject without any underlying condition before COVID-19, however, was diagnosed with ACOS (asthma and COPD overlap syndrome) at the 16-month follow-up. The proportion of patients with dyspnoea, defined as an MRC dyspnoea grade 2 or more, decreased from 21.0% (21/100) at 6 months to 9.1% (1/11) at 16 months, which was lower than the baseline proportion before infection of 16.7% (18/186) (Table 3).

## Discussion

This is the first report on longitudinal health outcomes after SARS-CoV-2 infection in Vietnam which included details of the clinical and epidemiological features of SARS-CoV-2 infection during the first domestic outbreak in Da Nang, Vietnam. In the retrospective part of our ambidirectional cohort study, we identified 362 individuals with laboratory-confirmed SARS-CoV-2, 7.8% with severe symptoms and a 5.5% mortality rate. Compared to the survivors, a significantly greater proportion of fatal cases were older and had preexisting conditions before their SARS-CoV-2 infection. In our prospective follow-up surveys, a noticeable proportion of participants had at least one symptom within seven days, 72.0% at 6 months, which slightly improved to 63.5% at 16 months. However, in the 16-month survey, more than 90% of the participants believed that they fully recovered from COVID-19, and 80% of the participants had returned to work. We also found that females and elderly individuals reported a significantly higher rate of long-term symptoms in the follow-up surveys.

The mortality rate in this study was 5.5% which was slightly higher than the mortality rate of 2.3% reported in the first outbreak in Wuhan, China [1]. This high mortality rate in our study might have been a reflection of the features of this outbreak, which began at the major hospital in Da Nang and then spread among hospitalised patients with preexisting conditions. In fact, almost all of the fatal cases in this study were hospitalised cases for preexisting conditions at the time of infection. The reported overall mortality rate of the Da Nang outbreak was 6.4% (35/551), which was slightly higher than that reported in this study [7]. This may suggest that the findings of this study could have reflected a high percentage of the overall features of the Da Nang outbreak.

Although the prevalence of longitudinal health problems in previous studies varies widely (10–70%) owing to the heterogeneity of those studies [1–4], the proportion of participants who reported at least one symptom in our follow-up surveys, 72.0% at 6 months and 63.5% at 16 months, was consistent with previous reports. A follow-up study in Wuhan, China [1], identified that the proportion of COVID-19 survivors with at least one sequelae symptom decreased significantly from 68% (777/1149) at 6 months to 55% (650/1190) at 2 years (P < 0.001). This study was conducted when the Wuhan strain was dominant, as it was during the period of our study. Additionally, their findings were consistent with our findings in several other aspects, such as the fact that approximately a half of the survivors experienced fatigue at 6 months or that sleeping problems were common symptoms in the follow-up surveys.

In our study, the prevalence of most symptoms decreased from the 6- to 16-month follow-up survey. In particular, the prevalence of coughing, which was the most common symptom, drastically decreased. However, the prevalence of some symptoms slightly increased at 16 months especially among females. The symptoms whose prevalence increased from 6 to 16 months in our study, including muscle pain and sleeping problems, showed a tendency of co-occurrence in a previous study involving symptom clustering analysis [20]. These findings could suggest that several different mechanisms of symptom development result in various longitudinal transitions of the symptoms.

In previous reports concerning the risk factors for long-COVID, increasing age and body mass index, female sex, type 2 diabetes, Epstein-Barr virus reactivation, the prevalence of specific autoantibodies, connective tissue disorders, attention deficit hyperactivity disorder, chronic urticaria and allergic rhinitis were potentially included [3, 6, 21, 22]. Our results also revealed that a significantly greater proportion of females and elderly individuals had at least one symptom in the follow-up surveys. A Russian group assumed that the genetic predisposing factors, including HLA types, the immunoregulatory functions of oestrogen and the expression of ACE-2, were associated with a higher rate of post COVID-19 syndrome among females [23]. The difference in symptom prevalence or co-occurrence between males and females could be due to the various pathophysiologies of long-COVID symptoms. Previous studies have investigated the effects of COVID-19 vaccination on long-COVID symptoms in various ways [24–26]. We compared symptom prevalence in the 16-month survey according to the doses of COVID-19 vaccination that the participants had received, and our results did not reveal a significant difference.

In our study, the proportion of individuals who reported anxiety or pain increased at 6 months after infection and then decreased to under preinfection levels at 16 months, with anxiety rates of 14.9% (11/74) before infection, 27% (20/74) at 6 months and 8.6% (5/58) at 16 months, and pain rates of 9.5% (7/74), 23.0% (17/74) and 6.9% (4/58), respectively. Although recall bias of the preinfection status could not be avoided, our results at least showed that the number of individuals with anxiety or pain at 16 months decreased compared to that at 6 months. These results were consistent with the reports from Switzerland [27]. Their results revealed that the proportion of participants with adverse outcomes started to decrease by one month after infection and became similar to preinfection levels by 24 months. They also reported that individuals who had anxiety before COVID-19 tended to have anxiety in the follow-up investigations. Although we did not have a comparison group and could not evaluate health status in the general population during this period, more than 90% of the participants agreed or strongly agreed that they felt fully recovered from COVID-19 at 16 months after infection, and 80% of the participants had returned to work. These results might suggest that the participants who experienced medical issues at the follow-up did not think that their medical issues were due to COVID-19 infection. The potential causes for feelings of anxiety could include damage to the nervous system, concerns about SARS-CoV-2 infection itself or sequelae, as well as the uncertain social situation caused by the pandemic.

Previous prospective studies on respiratory outcomes in patients following COVID-19 in China reported that in most patients who recovered from severe COVID-19, MRC dyspnoea scores and exercise capacity improved over time; however, some patients experienced persistent physiological and radiographic changes [1, 28]. The results of these studies included residual abnormalities in pulmonary function and a reduction in gas transfer, as measured by the diffusion capacity of carbon monoxide. Additionally, persistent radiological changes were observed in some patients, including findings that were potentially consistent with evolving fibrosis. Although, in our study, we had limited results in terms of MRC dyspnoea grade and pulmonary function tests, the prevalence of respiratory symptoms and the abnormal chest X-ray results improved in most participants at 16 months after infection. Our results concerning respiratory outcomes following SARS-CoV-2 infection were consistent with those of previous studies.

### Strengths and limitations

The strengths of our study are as follows. We enrolled a large proportion of SARS-CoV-2 infection cases in the outbreak in Da Nang regardless of their disease severity. This allowed us to describe the epidemiological and clinical features of the first domestic SARS-CoV-2 outbreak in Vietnam extensively. In addition, we conducted follow-up surveys until 16 months after infection, and this is the first report from Vietnam on longitudinal health outcomes among the SARS-CoV-2 survivors. Our study also has several limitations. Firstly, we could not avoid introducing information bias since the data were collected on the basis of self-reports. We did not have a comparison group, so we could not conclude whether the reported symptoms in the follow-up surveys were related to COVID-19. To address this issue, we asked participants about their health status before COVID-19 in the 6-month follow-up survey; however, this information was also based on the self-reports and could not avoid recall bias. Secondly, selection bias for the follow-up surveys could have been introduced since the individuals who had any symptoms or concerns might have been more motivated to participate in the follow-up surveys than those who did not have any symptoms or concerns. Additionally, the high proportion of participants lost to follow-up made it difficult to compare those who participated in the first follow-up and those in the second follow-up as longitudinal samples. Thirdly, we could not assess the impact of reinfections or COVID-19 vaccination on the increase in risk for long COVID sequelae, while multiple infections may cause additional harm [29]. In the follow-up surveys, asymptomatic COVID-19 reinfections were not investigated, however, no participants reported feverish COVID-19 reinfection histories within 3 months. Fourthly, with respect to pulmonary function tests, the available values and data were limited in our study. Total lung capacity was not tested in our study, and we could not differentiate between “mixed obstruction and restriction pulmonary disfunction” and “pure obstruction with pseudo restriction”. Since the lower limit of normal in Vietnamese people was not available, we used the “FEV1/FVC ratio” and “FVC percent of predicted” for interpreting pulmonary function tests, which were applied in previous references [17, 19]. Lastly, our findings cannot be expanded to infection with other variants since there can be differences in the prevalence of symptoms and long-term outcomes among variant strains.

## Conclusions

To the best of our knowledge, this is the first detailed report about the clinical and epidemiological features of the first domestic SARS-CoV-2 outbreak in Vietnam, including longitudinal health status observations of the survivors in this outbreak. In this study, a higher case fatality rate was observed than in other outbreaks of SARS-CoV-2. The prevalence of preexisting conditions was significantly greater among fatal cases than among survivors. A substantial proportion of the survivors reported long term health problems. Females and elderly individuals tended to have more problems, which might be due to the pathophysiological differences according to sex and age. Further investigations to elucidate the mechanisms and effective interventions for long COVID should be implemented.

## Data Availability

The datasets generated and analysed during the current study are available from the corresponding author on reasonable request.

## Abbreviations

ACOS: Asthma and COPD overlap syndrome
CES-D: Center for epidemiologic studies depression scale
COPD: Chronic obstructive pulmonary disease
EQ-5D-5L: 5-Level version of the EuroQol 5-dimenssion
EQ-VAS: EuroQol visual analogue scale
FEV1: Forced expiratory volume in one second
FVC: Forced vital capacity
IQR: Interquartile range
ISARIC: International severe acute respiratory and emerging infection consortium
MRC dyspnoea scale: Medical research council dyspnoea scale
real-time PCR: Real-time polymerase chain reaction
PFTs: Pulmonary function tests
SARS-CoV-2: Severe acute respiratory syndrome coronavirus 2
WG-SS: Washington group short set on functioning questions
